# Adherence to a Planetary Health Diet, Environmental Impacts, and Mortality in Chinese Adults

**DOI:** 10.1001/jamanetworkopen.2023.39468

**Published:** 2023-10-24

**Authors:** Yi-Xiang Ye, Ting-Ting Geng, Yan-Feng Zhou, Pan He, Ji-Juan Zhang, Gang Liu, Walter Willett, An Pan, Woon-Puay Koh

**Affiliations:** 1 Department of Epidemiology and Biostatistics, School of Public Health, Tongji Medical College, Huazhong University of Science and Technology, Wuhan, China; 2 Department of Nutrition and Food Hygiene, School of Public Health, Institute of Nutrition, Fudan University, Shanghai, China; 3 Department of Social Medicine and Health Management, School of Public Health, Guangxi Medical University, Nanning, China; 4 School of Earth and Environmental Sciences, Cardiff University, Cardiff, United Kingdom; 5 Department of Nutrition and Food Hygiene, Hubei Key Laboratory of Food Nutrition and Safety, Ministry of Education Key Lab of Environment and Health, and State Key Laboratory of Environmental Health (Incubating), School of Public Health, Tongji Medical College, Huazhong University of Science and Technology, Wuhan, China; 6 Harvard T.H. Chan School of Public Health, Harvard Medical School, Channing Division of Network Medicine, Brigham and Women’s Hospital, Boston, Massachusetts; 7 Healthy Longevity Translational Research Programme, Yong Loo Lin School of Medicine, National University of Singapore, Singapore; 8 Singapore Institute for Clinical Sciences, Agency for Science Technology and Research, Singapore

## Abstract

**Importance:**

Although the EAT-Lancet Commission has recently proposed a planetary health diet (PHD) to promote human and environmental health, little is known about how PHD affects environment and mortality risk among an Asian population.

**Objective:**

To investigate whether a PHD score is associated with environmental impacts and mortality outcomes in a Chinese cohort living in Singapore.

**Design, Setting, and Participants:**

This cohort study used data from the Singapore Chinese Health Study. Eligible participants were without known cardiovascular disease and cancer at baseline; they were recruited between 1993 and 1998 and followed up using record linkage data until 2020. Data were analyzed from September 2022 to April 2023.

**Exposures:**

PHD score was calculated based on the reference consumption of 14 dietary components in PHD and individual energy intake assessed using a validated food frequency questionnaire in this cohort.

**Main Outcomes and Measures:**

Diet-related environmental impacts were estimated using a food frequency questionnaire. Mortality outcomes (all-cause, cardiovascular disease, cancer, and respiratory disease) were identified via linkage with a nationwide registry.

**Results:**

A total of 57 078 participants were included in this study (mean [SD] age, 56.1 (7.9) years; 31 958 women [56.0%]). During a median (IQR) follow-up of 23.4 (18.7-26.2) years, 22 599 deaths occurred. Comparing the highest and lowest quintiles, higher PHD scores were associated with lower greenhouse gas emissions (β = −0.13 kg CO_2_ equivalent; 95% CI, −0.14 to −0.12 kg CO_2_ equivalent), but with higher total water footprint (β = 0.12 m^3^; 95% CI, 0.11-0.13 m^3^) and land use (β = 0.29 m^2^; 95% CI, 0.28-0.31 m^2^). In the adjusted multivariable model, compared with the lowest quintile, participants in the highest quintile of PHD score had lower risk of all-cause mortality (hazard ratio [HR], 0.85; 95% CI, 0.81-0.89), cardiovascular disease mortality (HR, 0.79; 95% CI, 0.73-0.85), cancer mortality (HR, 0.93; 95% CI, 0.86-1.00), and respiratory disease mortality (HR, 0.81; 95% CI, 0.74-0.89).

**Conclusions and Relevance:**

In this study of Singapore Chinese adults, higher adherence to PHD was associated with reduced risk of chronic disease mortality. However, environmental impacts were uncertain, as higher adherence was associated with lower greenhouse gas emissions but higher total water footprint and land use.

## Key Points

**Question:**

Is adherence to the planetary health diet associated with measurable impact on environmental sustainability and lower the risk of mortality?

**Findings:**

In this cohort study including 57 078 Singapore Chinese adults, higher adherence to the planetary health diet was associated with a lower risk of chronic disease mortality. For estimated environmental impacts, higher adherence was associated with lower greenhouse gas emissions, but higher total water footprint and land use.

**Meaning:**

These results suggest that adherence to the planetary health diet in a Chinese population may be beneficial for improving health outcomes, although the benefit on environment is less certain.

## Teaser

This cohort study examines the association of a planetary health diet with estimated environmental impacts and chronic disease mortality among Singapore Chinese adults.

## Introduction

The food system is closely related to human health.^1^ Unhealthy diets, such as those rich in red meat and ultra-processed food, contribute to increased risks of type 2 diabetes, cardiovascular diseases (CVD), and other noncommunicable diseases worldwide.^2,3^ However, the impacts of food systems are not limited to human health. Food production is reported to be a major driver of global environmental changes.^4^ Agriculture accounts for up to 30% of greenhouse gas emissions (GHG), 70% of freshwater use, and nearly 50% of land use, and livestock for the production of animal-based foods is a major contributor to these environmental impacts related to agriculture.^4,5,6^ Thus, a global transformation of diets could cobenefit human and planetary health.

In 2019, the EAT-Lancet Commission proposed a universal win-win healthy diet to address human and environmental health.^6^ This planetary health diet (PHD) advocated higher intake of plant-based foods and limited consumption of animal-sourced foods. Although several studies have developed scoring methods to assess adherence to PHD, no consensus has been formed. Moreover, these studies were conducted only in Western populations, without considering each individual’s energy intake^7,8,9,10,11^ and different levels of adherence to PHD.^12,13^ To date, only a few studies using individualized data have linked PHD to both environmental impacts and mortality outcomes.^10,14,15^ Laine et al^14^ revealed that higher adherence to PHD was associated with lower GHG emissions, land use, and mortality risk among Europeans. Another study conducted in the same cohort showed similar results but observed increased blue water use.^10^ In addition, Guo et al^15^ conducted a study on only some of the dietary components of PHD and found that shifting to PHD reduced premature death but increased GHG emissions and water use in China.

Given that findings on the associations of PHD with environmental impacts and mortality remain inconsistent, we aimed to develop a scoring method to measure adherence to PHD and to investigate the potential benefits from PHD for environment and health in a Chinese population living in Singapore.

## Methods

### Study Population

Data used in this study were from the Singapore Chinese Health Study (SCHS), which was a population-based prospective cohort study launched between 1993 and 1998. A total of 63 257 Chinese adults aged 45 to 74 years were recruited at baseline. The participants were permanent residents or citizens of Singapore who belonged to the Hokkien or Cantonese dialect group that originated either from the Fujian or Guangdong province in the southern part of China, respectively.^16^

For the current study, we excluded participants who had implausible energy intake (under 600 or above 3000 kcal/d for women and under 700 or above 3700 kcal/d for men; 1060 participants), and those who had self-reported diagnosis of cardiovascular diseases (3401 participants) or cancer at baseline (1718 participants), leaving 57078 participants for analysis (eFigure 1 in Supplement 1). The study was approved by the institutional review board of the National University of Singapore, and all participants gave written informed consent at enrollment. This study followed the Strengthening the Reporting of Observational Studies in Epidemiology (STROBE) reporting guideline.

### Calculation of the PHD Score (PHD-S)

Scoring for the PHD-S was constructed using a validated food frequency questionnaire based on the EAT-Lancet report (eMethods 1 and eTable 1 in Supplement 1).^6^ All 14 dietary components were classified into 3 categories based on their health effects: adequacy, optimum, and moderation.^17^ In the current study, vegetables, fruits, nuts, legumes, unsaturated fats, and fish were defined as adequacy components. Potatoes, dairy, poultry, and eggs were classified as optimum components. Total grains, red meat, saturated fats, and added sugar were considered as moderation components (eTable 2 in Supplement 1). The score for each dietary component ranged from 0 to 10 and the total PHD-S ranged from 0 to 140 (highest adherence) (eMethods 2 and eTable 2 in Supplement 1). For the purpose of computing the PHD-S, we standardized the energy intake of individuals to 2500 kcal/d.

### Estimation of Environmental Impacts

GHG emissions, total water footprint (TWF), and land use were estimated from the daily dietary intake of the participants using a conversion database from a study that investigated the environmental impacts of diet in the China Health and Nutrition Survey.^18^ Briefly, the environmental impact of the daily diet for individuals was calculated by multiplying the average environmental impact per gram of food by the amount of food consumed. GHG emissions were estimated from field to farm gate (impact of food production until the food was ready for consumption). TWF for nonaquatic foods was calculated using the database of the Water Footprint Network, and that for aquatic foods was calculated following the method in a previous study.^19^ Land use was estimated using data from the Food and Agriculture Organization Statistics database. GHG emissions were expressed as kilogram CO_2_ equivalents, TWF as meters cubed, and land use as meters squared.

### Assessment of Covariates and Mortality

At baseline, face-to-face interviews were conducted by trained interviewers using a structured questionnaire to collect information. More detailed information on covariates is available in eMethods 3 in Supplement 1. Deaths from CVD, cancer, and respiratory disease were coded according to the *International Classification of Diseases, Ninth Revision (ICD-9)* up to December 31, 2011, or *ICD-10* from 2012 to 2020, and ascertained via linkage with the Singapore Registry of Births and Deaths through December 31, 2020 (eMethods 4 in Supplement 1).

### Statistical Analysis

Linear regression models adjusted for age at baseline (years), sex (men or women), and total energy intake (kcal/d) were used to estimate the associations between PHD-S and environmental impacts. Person-years were calculated from the date of recruitment to either the date of death, loss to follow-up, or December 31, 2020, whichever came first. Cox proportional hazards regression models were used to estimate the hazard ratios (HRs) with corresponding 95% CIs for the associations of PHD-S with the risk of mortality using the lowest quintile of PHD-S as the reference group. The Schoenfeld residuals method was used to test the proportionality assumption of the Cox models and no violation was observed. In model 1, we adjusted for age, sex, and total energy intake. In model 2, we additionally adjusted for dialect group (Cantonese or Hokkien), educational level (no formal education, primary school, or secondary school or higher), BMI (continuous; calculated as weight in kilograms divided by height in meters squared), smoking status (never, former, or current), alcohol frequency (never, monthly, weekly, or daily), moderate or vigorous physical activity level (under 0.5, 0.5 to 3.9, or 4.0 or more h/wk), sleep duration (under 6, 6 to 8, or 8 h/d), and self-reported history of physician-diagnosed hypertension and diabetes. Linear trends were tested by using the median PHD-S within each quintile.

We repeated analyses stratified by age (younger than 55 years or 55 years and older), sex (using sex-specific quintiles for PHD-S in men and women), BMI (under 23 or 23 and above),^20^ and smoking status (never or ever) to test potential variation in associations among different subpopulations. Likelihood ratio test was used to examine the potential interaction. In sensitivity analysis, we first excluded participants with history of hypertension or diabetes at baseline. Second, we excluded participants who died within 5 years from recruitment to minimize the potential reverse causality. Third, we repeated the analyses with PHD-S calculated using the method proposed by Knuppel.^12^

The statistical analyses were conducted using Stata/MP version 17.0 (StataCorp LLC). A 2-sided *P* < .05 was considered statistically significant. Data analysis was performed from September 2022 to April 2023.

## Results

### Baseline Characteristics

Of the 57 078 participants in the SCHS, 31 958 (56.0%) were women, and the median (IQR) age was 55.0 (49.0-62.0) years. Participants with higher adherence to PHD were younger (median [IQR] age: quintile 5, 54.0 [48.0-60.0] years vs quintile 1, 57.0 [51.0-64.0] years), more likely to be women (7514 of 11 415 [65.8%] vs 5352 of 11 416 [46.9%]), more highly educated (secondary school or higher: 4178 [36.6%] vs 2281 [19.9%]), never smokers (9212 [80.7%] vs 6409 [56.2%]), nondrinkers (9533 [83.5%] vs 8785 [77.0%]), and physically active (less than 0.5 h/wk: 6956 [60.9%] vs 8234 [72.1%]) (Table 1).

**Table 1.** Baseline Characteristics of the Study Population by Quintiles of the Planetary Healthy Diet Score in the Singapore Chinese Health Study

Characteristic	Planetary health diet score quintiles, patients, No. (%)				
	Quintile 1 (n = 11 416)	Quintile 2 (n = 11 416)	Quintile 3 (n = 11 415)	Quintile 4 (n = 11 416)	Quintile 5 (n = 11 415)
Planetary health diet score, median (IQR)	43 (40-46)	50 (49-52)	55 (54-57)	60 (59-62)	67 (65-71)
Age, median (IQR), y	57.0 (51.0-64.0)	56.0 (50.0-63.0)	55.0 (49.0-62.0)	54.0 (49.0-61.0)	54.0 (48.0-60.0)
Sex					
Men	6064 (53.1)	5589 (49.0)	5000 (43.8)	4566 (40.0)	3901 (34.2)
Women	5352 (46.9)	5827 (51.0)	6415 (56.2)	6850 (60.0)	7514 (65.8)
BMI, median (IQR)	23.0 (20.9-24.1)	23.11 (21.1-24.6)	23.1 (21.1-24.8)	23.1 (21.2-24.8)	23.1 (21.1-24.8)
Educational level					
No formal education	3957 (34.7)	3126 (27.4)	2967 (26.0)	2750 (24.1)	2437 (21.4)
Primary school	5178 (45.4)	5217 (45.7)	5146 (45.1)	5039 (44.1)	4800 (42.0)
Secondary school or higher	2281 (19.9)	3073 (26.9)	3302 (28.9)	3627 (31.8)	4178 (36.6)
Dialect group					
Cantonese	4405 (38.6)	5157 (45.2)	5321 (46.6)	5594 (49.0)	5940 (52.0)
Hokkien	7011 (61.4)	6259 (54.8)	6094 (53.4)	5822 (51.0)	5475 (48.0)
Smoking status					
Never	6409 (56.2)	7514 (65.8)	8251 (72.3)	8658 (75.8)	9212 (80.7)
Former	1258 (11.0)	1326 (11.6)	1160 (10.2)	1094 (9.6)	986 (8.6)
Current	3749 (32.8)	2576 (22.6)	2004 (17.5)	1664 (14.6)	1217 (10.7)
Alcohol frequency					
None	8785 (77.0)	9049 (79.3)	9261 (81.1)	9344 (81.9)	9533 (83.5)
Monthly	812 (7.1)	871 (7.6)	875 (7.7)	873 (7.6)	881 (7.7)
Weekly	1086 (9.5)	1061 (9.3)	951 (8.3)	905 (7.9)	774 (6.8)
Daily	733 (6.4)	435 (3.8)	328 (2.9)	294 (2.6)	227 (2.0)
Physical activity					
<0.5 h/wk	8234 (72.1)	7745 (67.8)	7615 (66.7)	7595 (66.5)	6956 (60.9)
0.5-3.9 h/wk	1785 (15.6)	2202 (19.3)	2358 (20.7)	2311 (20.2)	2763 (24.2)
≥4 h/wk	1397 (12.3)	1469 (12.9)	1442 (12.6)	1510 (13.3)	1696 (14.9)
Sleep duration					
<6 h/d	1123 (9.8)	1030 (9.0)	1020 (8.9)	1007 (8.8)	1120 (9.8)
6-8 h/d	9389 (82.3)	9670 (84.7)	9648 (84.5)	9669 (84.7)	9641 (84.5)
>8 h/d	904 (7.9)	716 (6.3)	747 (6.6)	740 (6.5)	654 (5.7)
History of hypertension	2193 (19.2)	2412 (21.1)	2545 (22.3)	2600 (22.8)	2674 (23.4)
History of diabetes	654 (5.7)	837 (7.3)	891 (7.8)	1034 (9.1)	1078 (9.4)
GHGe, median (IQR), kg CO_2_	2.8 (2.6-3.0)	2.7 (2.5-2.9)	2.7 (2.5-2.9)	2.7 (2.5-2.9)	2.6 (2.4-2.8)
TWF, median (IQR), m^3^	2.4 (2.3-2.6)	2.5 (2.3-2.7)	2.5 (2.4-2.7)	2.5 (2.4-2.7)	2.6 (2.4-2.7)
Land use, median (IQR), m^2^	2.9 (2.6-3.3)	3.0 (2.7-3.4)	3.1 (2.8-3.4)	3.1 (2.8-3.5)	3.2 (2.9-3.7)

Abbreviations: BMI, body mass index (calculated as weight in kilograms divided by height in meters squared); GHGe, greenhouse gas emissions; TWF, total water footprint.

This Singapore Chinese population consumed more fruits, fish, grains (mostly refined), red meat, and saturated fats and less vegetables, nuts, legumes, unsaturated fats, sugar, potatoes, dairy, and poultry than what was recommended by the PHD (eTable 3; eFigure 2 in Supplement 1). The PHD-S ranged between 13 and 95 points, with a median of 55 points (eFigure 3 in Supplement 1). Adherence to PHD was therefore considered low in this study population, and more than 80% of respondents had good compliance (more than 6 points for each dietary component) in only 3 out of 14 components, including fruits, unsaturated fats, and fish (eFigure 4 in Supplement 1).

### PHD-S and Environmental Impacts

Among all the participants, the estimated median (IQR) GHG emissions, TWF, and land use from daily food consumption were 2.7 (2.5-2.9) kg CO_2_ equivalents, 2.5 (2.4-2.7) m^3^, and 3.1 (2.8-3.5) m^2^, respectively. Compared with the lowest quintile of PHD-S, the highest quintile had reduced GHG emissions by 7.1% (median [IQR]: Q5, 2.6 [2.4-2.8] kg CO_2_ equivalent vs Q1, 2.8 [2.6-3.0] kg CO_2_ equivalent), and increased TWF by 8.3% (2.6 [2.4-2.7] m^3^ vs 2.4 [2.3-2.6] m^3^) and land use by 10.3% (3.2 [2.9-3.7] m^2^ vs 2.9 [2.6-3.3] m^2^) (Table 1). Higher quintiles of PHD-S were associated in a stepwise manner with lower GHG emissions (β = −0.13 kg CO_2_ equivalent; 95% CI, −0.14 to −0.12 kg CO_2_ equivalent), higher TWF (β = 0.12 m^3^; 95% CI, 0.11 to 0.13 m^3^), and more land use (β = 0.29 m^2^; 95% CI, 0.28 to 0.31 m^2^) (all *P* for trend < .001) (Table 2).

**Table 2.** Regression Coefficients for the Association Between Quintiles of the Planetary Health Diet Score and Environmental Indicators^a^

Indicators	Median (IQR)	Quintiles of the planetary health diet score (range)					*P* value for trend^b^
		Quintile 1 (13-47)	Quintile 2 (47-53)	Quintile 3 (53-58)	Quintile 4 (58-63)	Quintile 5 (63-95)	
GHGe, kg CO_2_	2.7 (2.5 to 2.9)	0 [Reference]	−0.04 (−0.05 to −0.03)	−0.06 (−0.07 to −0.05)	−0.08 (−0.09 to −0.07)	−0.13 (−0.14 to −0.12)	<.001
Total water footprint, m^3^	2.5 (2.4 to 2.7)	0 [Reference]	0.06 (0.05 to 0.07)	0.08 (0.08 to 0.09)	0.10 (0.09 to 0.10)	0.12 (0.11 to 0.13)	<.001
Land use, m^2^	3.1 (2.8 to 3.5)	0 [Reference]	0.12 (0.11 to 0.14)	0.16 (0.15 to 0.18)	0.20 (0.19 to 0.22)	0.29 (0.28 to 0.31)	<.001

Abbreviation: GHGe, greenhouse gas emissions.

^a^ Models were adjusted for age, sex, and total energy intake.

^b^ Linear trends were assessed by treating the median values of the quintiles of planetary health diet score as a continuous variable.

**Figure 1.**
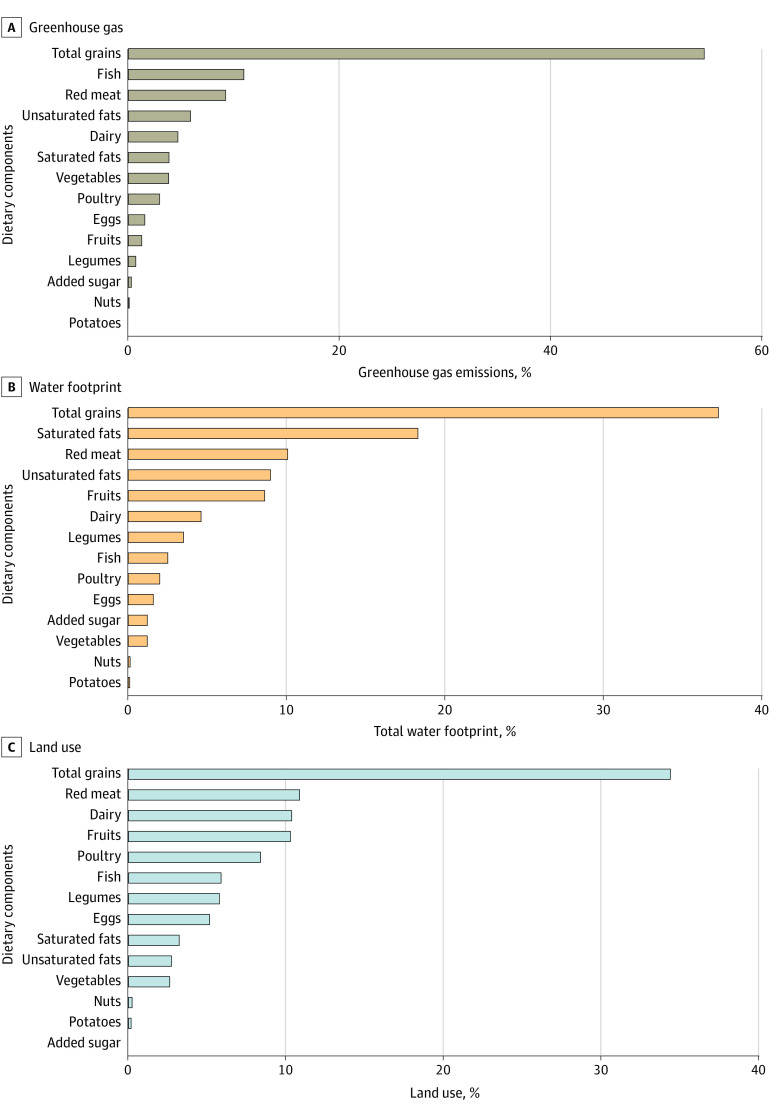
Estimated Contributions of Each Dietary Component in the Planetary Health Diet to Greenhouse Gas Emissions, Total Water Footprint, and Land Use

**Figure 2.**
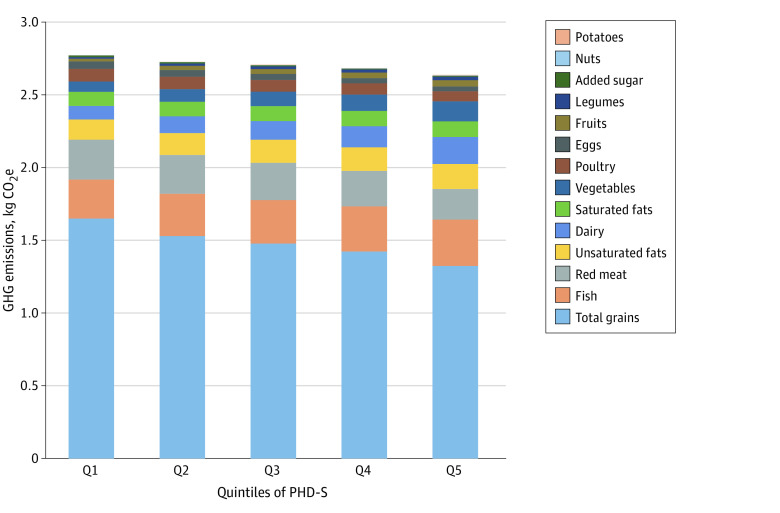
GHG Emissions Across Quintiles of the Planetary Health Diet Score From Different Dietary Components CO_2_e indicates CO_2_ equivalent; GHG, greenhouse gas emissions; PHD-S, planetary health diet score.

The dietary components that contributed the most to GHG emissions included total grains (54.61%), fish (10.95%), and red meat (9.22%) (Figure 1). Plant-based food that contributed the most to TWF and land use was total grains (37.25% of TWF and 34.39% of land use), followed by fruits, which was responsible for 8.61% of TWF and 10.28% of land use. Red meat was also resource-intensive and was responsible for 10.07% of TWF and 10.86% of land use. Aside from red meat, animal-based food with the highest land use included dairy (10.35%), poultry (8.38%), and fish (5.87%) (Figure 1). Higher PHD-S was associated with overall lower GHG emissions, and this was largely explained by decreased contribution of GHG emissions from reduced consumption of total grains (quintile 5, 1.32 kg CO_2_ equivalent vs quintile 1, 1.65 kg CO_2_ equivalent) and red meat (quintile 5, 0.21 kg CO_2_ equivalent vs quintile 1, 0.27 kg CO_2_ equivalent) in higher PHD-S quintiles (Figure 2; eTable 4 in Supplement 1). In contrast, higher PHD-S was associated with overall higher TWF and land use, and this was mainly due to increased contribution of TWF and land use from greater consumption of fruits, dairy, vegetables, and legumes in higher PHD-S quintiles (eFigures 5 and 6 and eTables 5 and 6 in Supplement 1).

### Association of PHD-S With Mortality

During 1 211 192 person-years of follow-up (median [IQR] follow-up, 23.4 [18.7-26.2] years), a total of 22 599 deaths were documented (39.6%), including 6948 CVD deaths (3830 from ischemic heart disease, 1866 from stroke), 7338 cancer deaths, and 4891 respiratory disease deaths [3944 from pneumonia, 807 from chronic obstructive pulmonary disease (COPD)] (Table 3; and eTable 7 in Supplement 1).

**Table 3.** Association Between Planetary Health Diet Score and All-Cause and Cause-Specific Mortality in the Singapore Chinese Health Study

Variable	Quintiles of planetary health diet score, HR (95% CI)					*P* value for trend^a^
	Quintile 1	Quintile 2	Quintile 3	Quintile 4	Quintile 5	
Planetary health diet score, range	13-47	47-53	53-58	58-63	63-95	NA
Person-year of follow-up	228 222	239 839	244 012	247 935	251 184	NA
All-cause mortality						
Events, No.	5648	4712	4412	4147	3680	NA
Model 1^b^	1 [Reference]	0.85 (0.82-0.89)	0.82 (0.79-0.86)	0.81 (0.77-0.84)	0.77 (0.74-0.80)	<.001
Model 2^c^	1 [Reference]	0.90 (0.87-0.94)	0.89 (0.85-0.92)	0.88 (0.84-0.91)	0.85 (0.81-0.89)	<.001
**Cause-specific mortality**						
CVD mortality						
Events, No.	1771	1403	1387	1254	1133	NA
Model 1^b^	1 [Reference]	0.82 (0.76-0.88)	0.84 (0.78-0.90)	0.79 (0.73-0.85)	0.77 (0.71-0.83)	<.001
Model 2^c^	1 [Reference]	0.84 (0.78-0.90)	0.85 (0.79-0.92)	0.81 (0.75-0.87)	0.79 (0.73-0.85)	<.001
Cancer mortality						
Events, No.	1810	1552	1409	1360	1207	NA
Model 1^b^	1 [Reference]	0.87 (0.81-0.93)	0.81 (0.76-0.87)	0.81 (0.75-0.87)	0.76 (0.71-0.82)	<.001
Model 2^c^	1 [Reference]	0.96 (0.89-1.03)	0.93 (0.86-0.99)	0.95 (0.88-1.02)	0.93 (0.86-1.00)	.04
Respiratory diseases mortality						
Events, No.	1274	1052	943	883	739	NA
Model 1^b^	1 [Reference]	0.84 (0.77-0.91)	0.79 (0.72-0.85)	0.78 (0.71-0.85)	0.71 (0.65-0.78)	<.001
Model 2^c^	1 [Reference]	0.90 (0.83-0.98)	0.87 (0.80-0.95)	0.87 (0.80-0.95)	0.81 (0.74-0.89)	<.001

Abbreviations: CVD, cardiovascular disease; NA, not applicable.

^a^ Linear trends were assessed by treating the median values of the quintiles of planetary health diet score as a continuous variable.

^b^ Model 1 was adjusted for age, sex, and energy intake (kcal/d).

^c^ Model 2 was additionally adjusted for dialect group (Cantonese or Hokkien), educational level (no formal education, primary school, or secondary school or higher), body mass index, smoking status (never, former, or current), alcohol frequency (none, monthly, weekly, or daily), physical activity (<0.5 h/wk, 0.5-3.9 h/wk, or ≥4 h/wk), sleep duration (<6 h/d, 6-8 h/d, or >8 h/d), and self-reported history of physician-diagnosed hypertension and diabetes.

In model 1, higher PHD-S was significantly associated with lower risk of all-cause, CVD, cancer, and respiratory disease mortality (all *Ps* for trend <.001) (Table 3). The associations were attenuated but remained significant after additional adjustment for other potential confounders. In model 2, participants in the highest quintile of PHD score had lower risk of all-cause mortality (HR, 0.85; 95% CI, 0.81-0.89; *P* < .001), CVD mortality (HR, 0.79; (95% CI, 0.73-0.85; *P* < .001), cancer mortality (HR, 0.93; 95% CI, 0.86-1.00; *P* = .04), and respiratory disease mortality (HR, 0.81; 95% CI, 0.74-0.89; *P* < .001) (Table 3).

We also estimated the associations between PHD-S and subtypes of mortality from CVD and respiratory disease. Participants in the highest quintile, compared with those in the lowest quintile, had an 11% to 35% lower risk of ischemic heart disease, stroke, pneumonia, and COPD mortality (eTable 7 in Supplement 1).

The stepwise reduction in mortality risk with increasing quintile of PHD-S was greater for individuals who had ever smoked than in those who had never smoked (*P* for interaction = .01); the HR comparing extreme quintiles was 0.80 (95% CI, 0.75-0.86) in ever-smokers vs 0.86 (95% CI, 0.81-0.91) in never-smokers. The stepwise reduction in mortality risk with increasing sex-specific quintile of PHD-S was higher in women than in men (*P* for interaction = .04); HR between extreme quintiles was 0.86 (95% CI, 0.81-0.91) in men vs 0.83 (95% CI, 0.78-0.88) in women (eTable 8 in Supplement 1). In sensitivity analyses, the results remained materially unchanged (eTables 9 and 10 in Supplement 1).

## Discussion

In this large population-based prospective cohort of Singapore Chinese adults, we developed a new scoring method to measure adherence to PHD, and observed inverse associations of PHD-S with the risk of all-cause and cause-specific mortality. However, adherence to PHD was associated with reduced GHG emissions but increased TWF and land use. Our findings suggest that adherence to PHD could benefit health, although the benefits on environment were less certain.

Our findings of the inverse associations of PHD with all-cause and cause-specific mortality were consistent with previous studies. Based on food availability data, the EAT-Lancet report found that the adoption of PHD could avoid 11.1 million deaths per year and reduce 19% of premature mortality by 2030.^6^ Similarly, Springman et al^21^ found that adherence to PHD was associated with a 19% to 22% reduction in premature mortality in a modeling analysis. This association was also observed in studies based on individualized data. The Malmö Diet and Cancer cohort study indicated a 25% reduction in all-cause mortality among those with the highest adherence to PHD, and the European Prospective Investigation into Cancer and Nutrition (EPIC) study estimated that up to 19% to 63% of deaths in this Europe-wide cohort could be prevented by the adoption of PHD.^13,14^ However, Knuppel et al^12^ did not observe a significant association between adherence to PHD and mortality among 46 069 participants in the EPIC-Oxford cohort. These inconsistencies in findings among different studies may be due to the differences in scoring methods and study populations. For example, Knuppel et al developed the PHD-S based on 14 food items with binary value (0 or 1 point) for each item, thus a narrower range of scores (0 to 14 points).^12^ On the other hand, we assigned 10 points to each food item and thus could cover a wider range of variations in food consumption. Moreover, the EPIC included participants from different cohorts recruited across 10 European countries, and the variance in the dietary profiles was large.^22^ To our knowledge, this study is the first to find an inverse association between adherence to PHD and the risk of respiratory disease mortality, especially mortality from COPD. Although the risk estimates in the fully adjusted model 2, which included strong risk factors for COPD such as smoking and body mass index,^23,24,25^ were substantially attenuated compared with those in the minimally adjusted model 1, the risk estimates were still statistically significant. This suggests that even after accounting for smoking and body mass index, improving diet quality could still have beneficial health effects for respiratory disease mortality.

We found that higher PHD-S was associated with lower GHG emissions, which was in line with other studies.^7,10,14^ This association was largely driven by lower consumption of total grains (mostly refined grains) and red meat, and was consistent with the findings that rice, as the main crop on a global scale, emits more GHG than other crops, and shifting to a diet free of animal products (especially red meat) could reduce GHG emissions by 49%.^26,27^ In the current study, higher intakes of fruits, dairy, vegetables, and legumes raised the PHD-S but also contributed to increased TWF and land use. In contrast, reduced consumption of red meat and total grains raised the PHD-S but contributed to decreased TWF and land use. As such, we recommend a reduction in the intake of total grains and red meat to offset the environmental impacts on TWF and land use from increased consumption of the recommended dietary components.

Furthermore, we observed that inverse association of PHD with mortality was stronger among individuals who had ever smoked than those who had never smoked, which may be explained by antioxidant nutrients in vegetables and fruits in PHD providing greater benefit for smokers than for never-smokers.^28^ Although we observed a greater stepwise reduction in mortality with increasing PHD-S in women than in men, the absolute difference in HR was small. Further studies would be needed to verify differential effects of PHD-S on mortality between men and women.

### Limitations

This study had several limitations. First, the dietary data was only collected at baseline and subsequent changes of dietary intake during follow-up were not measured. However, given the prospective design, these changes may likely lead to nondifferential misclassification and underestimation of associations. Second, the methods used to evaluate the environmental impacts in the current study were derived from a previous study conducted in China,^18^ which might not be generalizable to Singapore. Third, given that several of the covariates (age, sex, education, and smoking) had associations with PHD-S, the association between PHD-S and mortality was susceptible to confounding and, as expected, the effect size of the associations was attenuated after further adjustment for potential confounders. Hence, the results of this study and causal inferences should be interpreted with caution as this study is observational in design and residual confounders cannot be completely ruled out. Fourth, as the study participants were Singapore Chinese, it might limit the generalizability of the findings to other populations.

## Conclusions

Our findings suggest that adherence to PHD may be beneficial for reducing mortality risk and GHG emissions but may increase the TWF and land use among Singapore Chinese. More studies should be conducted in other populations to determine the cobenefit for human and environmental health so as to establish unequivocal evidence that supports a worldwide implementation of PHD.

## Notes

Supplement 1. **eAppendix 1.** Dietary Assessment **eAppendix 2.** Calculation of the Planetary Health Diet Score **eAppendix 3.** Assessment of Covariates **eAppendix 4.** Ascertainment of Mortality **eTable 1.** Healthy Reference Diet, With Possible Ranges, for an Intake of 2500 Kcal/day **eTable 2.** Construction of the Planetary Health Diet Score in the Singapore Chinese Health Study **eTable 3.** Median Intake and Complete Adherence of Dietary Components by Quintiles of the Planetary Health Diet Score from the Singapore Chinese Health Study **eTable 4.** GHG Emissions Across Quintiles of the Planetary Health Diet Score from Different Dietary Components **eTable 5.** TWF Across Quintiles of the Planetary Health Diet Score from Different Dietary Components **eTable 6.** Land Use Across Quintiles of the Planetary Health Diet Score from Different Dietary Components **eTable 7.** Hazard Ratios and 95% Confidence Intervals for the Association Between Planetary Health Diet Score and Subtypes of Cardiovascular Disease Mortality and Respiratory Disease Mortality in the Singapore Chinese Health Study **eTable 8.** Subgroup Analyses of the Association Between Planetary Health Diet Score and All-Cause Mortality in the Singapore Chinese Health Study **eTable 9.** Sensitivity Analyses of Multivariable-Adjusted Association of Planetary Health Diet Score With All-Cause and Cause-Specific Mortality in the Singapore Chinese Health Study **eTable 10.** Multivariable-Adjusted Association Between Previously Established Planetary Health Diet Score and All-Cause and Cause-Specific Mortality in the Singapore Chinese Health Study **eFigure 1.** Flow Chart of Final Sample From the Singapore Chinese Health Study **eFigure 2.** Comparison of the Recommended Range of the Planetary Health Diet and Median Intake of the Participants in the Singapore Chinese Health Study **eFigure 3.** Distribution of Planetary Health Diet Score in the Singapore Chinese Health Study **eFigure 4.** Compliance with Planetary Health Diet According to Score for Each Dietary Component **eFigure 5.** Total Water Footprint Across Quintiles of the Planetary Health Diet Score From Different Dietary Components **eFigure 6.** Land Use Across Quintiles of the Planetary Health Diet Score from Different Dietary Components
eReferences.

Supplement 2.
Data Sharing Statement
