# Supplementary material for: Adherence to a Planetary Health Diet, Environmental Impacts, and Mortality in Chinese Adults
Source: JAMA Netw Open. 2023 Oct 24;6(10):e2339468. doi: 10.1001/jamanetworkopen.2023.39468 (PMC10599124; doi:10.1001/jamanetworkopen.2023.39468)
Supplement: Supplement 1. — eAppendix 1. Dietary Assessment eAppendix 2. Calculation of the Planetary Health Diet Score eAppendix 3. Assessment of Covariates eAppendix 4. Ascertainment of Mortality eTable 1. Healthy Reference Diet, With Possible Ranges, for an Intake of 2500 Kcal/day eTable 2. Construction of the Planetary Health Diet Score in the Singapore Chinese Health Study eTable 3. Median Intake and Complete Adherence of Dietary Components by Quintiles of the Planetary Health Diet Score from the Singapore Chinese Health Study eTable 4. GHG Emissions Across Quintiles of the Planetary Health Diet Score from Different Dietary Components eTable 5. TWF Across Quintiles of the Planetary Health Diet Score from Different Dietary Components eTable 6. Land Use Across Quintiles of the Planetary Health Diet Score from Different Dietary Components eTable 7. Hazard Ratios and 95% Confidence Intervals for the Association Between Planetary Health Diet Score and Subtypes of Cardiovascular Disease Mortality and Respiratory Disease Mortality in the Singapore Chinese Health Study eTable 8. Subgroup Analyses of the Association Between Planetary Health Diet Score and All-Cause Mortality in the Singapore Chinese Health Study eTable 9. Sensitivity Analyses of Multivariable-Adjusted Association of Planetary Health Diet Score With All-Cause and Cause-Specific Mortality in the Singapore Chinese Health Study eTable 10. Multivariable-Adjusted Association Between Previously Established Planetary Health Diet Score and All-Cause and Cause-Specific Mortality in the Singapore Chinese Health Study eFigure 1. Flow Chart of Final Sample From the Singapore Chinese Health Study eFigure 2. Comparison of the Recommended Range of the Planetary Health Diet and Median Intake of the Participants in the Singapore Chinese Health Study eFigure 3. Distribution of Planetary Health Diet Score in the Singapore Chinese Health Study eFigure 4. Compliance with Planetary Health Diet According to Score for Each Dietary Component eFigure 5. Tot [file jamanetwopen-e2339468-s001.pdf]

Supplemental Online Content

Ye Y, Geng T, Zhou Y, et al. Adherence to a planetary health diet, environmental impacts, and mortality in Chinese adults. *JAMA Netw Open.* 2023;6(10):e2339468. doi:10.1001/jamanetworkopen.2023.39468

eAppendix 1. Dietary Assessment

eAppendix 2. Calculation of the Planetary Health Diet Score

eAppendix 3. Assessment of Covariates

eAppendix 4. Ascertainment of Mortality

eTable 1. Healthy Reference Diet, With Possible Ranges, for an Intake of 2500 kCal/day

eTable 2. Construction of the Planetary Health Diet Score in the Singapore Chinese Health Study

eTable 3. Median Intake and Complete Adherence of Dietary Components by Quintiles of the Planetary Health Diet Score from the Singapore Chinese Health Study

eTable 4. GHG Emissions Across Quintiles of the Planetary Health Diet Score from Different Dietary Components

eTable 5. TWF Across Quintiles of the Planetary Health Diet Score from Different Dietary Components

eTable 6. Land Use Across Quintiles of the Planetary Health Diet Score from Different Dietary Components

eTable 7. Hazard Ratios and 95% Confidence Intervals for the Association Between Planetary Health Diet Score and Subtypes of Cardiovascular Disease Mortality and Respiratory Disease Mortality in the Singapore Chinese Health Study

eTable 8. Subgroup Analyses of the Association Between Planetary Health Diet Score and All-Cause Mortality in the Singapore Chinese Health Study

eTable 9. Sensitivity Analyses of Multivariable-Adjusted Association of Planetary Health Diet Score With All-Cause and Cause-Specific Mortality in the Singapore Chinese Health Study

eTable 10. Multivariable-Adjusted Association Between Previously Established Planetary Health Diet Score and All-Cause and Cause-Specific Mortality in the Singapore Chinese Health Study

eFigure 1. Flow Chart of Final Sample From the Singapore Chinese Health Study

eFigure 2. Comparison of the Recommended Range of the Planetary Health Diet and Median Intake of the Participants in the Singapore Chinese Health Study

eFigure 3. Distribution of Planetary Health Diet Score in the Singapore Chinese Health Study

eFigure 4. Compliance with Planetary Health Diet According to Score for Each Dietary Component

© 2023 Ye Y et al. *JAMA Network Open.*

eFigure 5. Total Water Footprint Across Quintiles of the Planetary Health Diet Score From Different Dietary Components

eFigure 6. Land Use Across Quintiles of the Planetary Health Diet Score from Different Dietary Components

eReferences.

This supplemental material has been provided by the authors to give readers additional information about their work.

© 2023 Ye Y et al. *JAMA Network Open.*

eAppendix 1. Dietary Assessment

Detailed dietary intake over the past year was captured using a validated 165-item, semi-quantitative food frequency questionnaire (FFQ) by trained interviewers in the participants' homes. The FFQ was developed specifically for the Singapore Chinese Health Study (SCHS); the 8 response options for frequency of intake ranged from never or hardly ever to ≥2 times/day, and the portion sizes for most food items were estimated using colored photographs of three identical food items of various amounts (roughly representing the 15th, 50th, and 85th percentile intake) and actual plates to enable the participants to obtain a sense of scale. Daily nutrients and energy intake were calculated using the Singapore Food Composition Database developed for this cohort. The FFQ had been validated in a subset of 810 participants randomly selected from the SCHS.

© 2023 Ye Y et al. *JAMA Network Open.*

eAppendix 2. Calculation of the Planetary Health Diet Score

The 14 dietary components of the planetary health diet (PHD) were classified into 3 categories based on their health effects: adequacy, optimum, and moderation. Adequacy components are used to indicate healthy foods with higher recommended intakes. In the current study, vegetables, fruits, nuts, legumes, unsaturated fats, and fish were defined as adequacy components. Optimum components represent nutrient-dense foods but can be harmful if consumed in large amounts daily. Potatoes, dairy foods, poultry, and eggs were classified as optimum components. The moderation components are defined as those that could increase the risk of chronic diseases with increased consumption. As the majority of the total grains intake in this study population was refined grains, therefore, total grains, red meat, saturated fats, and added sugars were considered as moderation components in PHD.

For adequacy components, participants received 0 points for no consumption, 10 points for equal to or exceeding the cut-off values, while an intake from zero to cut-off values was proportionally scored between 0 and 10 points. For optimum components, an intake from zero to cut-off values was scored gradually from 0 to 10 points, while an intake from cut-off to threshold values was scored inversely, and an intake above threshold values was scored as 0. For moderation components, no consumption (saturated fats and added sugar) or less than cut-off values (red meat and total grain) were scored 10 points, while intake from zero or cut-off values to threshold values was scored gradually from 10 to 0 points. The scores for adequacy, optimum, and moderation components were calculated as follows, equation:

$$PHD\ score_a = \begin{cases} 0 \ \text{if} \ consumption_{ij} = 0 \\ \dfrac{consumption_{ij}}{cut-off_i} \times 10 \ \text{if} \ 0 < consumption_{ij} < cut-off_i \\ 10 \ \text{if} \ (consumption_{ij} - cut\text{-}off_i) \geq 0 \end{cases} \quad ①$$

a : adequacy component; i : refers to the 14 food groups; j : the individual participant in the study; PHD : planetary health diet.

$$PHD\ score_o = \begin{cases} 0 \ \text{if} \ consumption_{ij} = 0 \ \text{or} \ (consumption_{ij} - threshold\ value_i) \geq 0 \\ \dfrac{consumption_{ij}}{cut-off_i} \times 10 \ \text{if} \ 0 < consumption_{ij} < cut-off_i \\ 10 \ \text{if} \ consumption_{ij} = cut-off_i \\ 10 - \left(\dfrac{consumption_{ij} - cut-off_i}{threshold\ value_i - cut-off_i} \times 10 \right) \ \text{if} \ cut-off_i < consumption_{ij} < threshold\ value_i \end{cases} \quad ②$$

o : optimum component; i : refers to the 14 food groups; j : the individual participant in the study; PHD : planetary health diet.

$$PHD\ score_m = \begin{cases} 0 \ \text{if} \ (consumption_{ij} - threshold\ value_i) \geq 0 \\ 10 - \left(\dfrac{consumption_{ij} - cut-off_i}{threshold\ value_i - cut-off_i} \times 10 \right) \ \text{if} \ cut-off_i < consumption_{ij} < threshold\ value_i \\ 10 \ \text{if} \ consumption_{ij} = 0 \ \text{or} \ (cut-off_i - consumption_{ij}) \geq 0 \end{cases} \quad ③$$

m : moderation component; i : refers to the 14 food groups; j : the individual participant in the study; PHD : planetary health diet.

© 2023 Ye Y et al. *JAMA Network Open.*

eAppendix 3. Assessment of Covariates

At baseline, face-to-face interviews were conducted by trained interviewers using a structured questionnaire to collect information on demographics (age, sex, education, and dialect group), anthropometric data (height and weight), lifestyle factors (smoking status, alcohol consumption, sleep duration, and physical activity), and medical history (hypertension, coronary artery disease, stroke, diabetes, and cancer). Participants with a self-reported history of cancer at baseline were further ascertained by linking with the database of the nationwide Singapore Cancer Registry. Body mass index (BMI) was calculated as weight (kg) divided by the square of height (m^2).

© 2023 Ye Y et al. *JAMA Network Open.*

eAppendix 4. Ascertainment of Mortality

The International Classification of Diseases Ninth Revision (ICD-9) up to December 31, 2011, or Tenth Revision (ICD-10) from 2012 to 2020 were used to classify causes of death from CVD (ICD-9: 390–459; ICD-10: I00–I99), cancer (ICD-9: 140–208; ICD-10: C00–C97), and respiratory diseases (ICD-9: 460–519; ICD-10: J00–J99). Deaths from CVD were further specified as ischemic heart disease (ICD-9: 410–414; ICD-10: I20–25), and stroke (ICD-9: 430–438; ICD-10: I60–I69) that included hemorrhagic stroke (ICD-9: 430–432; ICD-10: I60–I62) and ischemic/non-specified stroke (ICD-9: 433–438; ICD-10: I63–I69). Deaths from respiratory diseases were also specified as pneumonia (ICD-9: 480–486; ICD-10: J12–J18), and chronic obstructive pulmonary disease (COPD) (ICD-9: 490–496; ICD-10: J40–J47).

© 2023 Ye Y et al. *JAMA Network Open.*

eTable 1. Healthy Reference Diet, with Possible Ranges, for An Intake of 2500 kcal/day

	Macronutrient intake (possible range), g/day	Caloric intake, kcal/day
Whole grains*		
Rice, wheat, corn, and other†	232 (total gains 0–60% of energy)	811
Tubers or starchy vegetables		
Potatoes and cassava	50 (0–100)	39
Vegetables		
All vegetables	300 (200–600)	···
Dark green vegetables	100	23
Red and orange vegetables	100	30
Other vegetables	100	25
Fruits		
All fruit	200 (100–300)	126
Dairy foods		
Whole milk or derivative equivalents (eg, cheese)	250 (0–500)	153
Protein sources‡		
Beef and lamb	7 (0–14)	15
Pork	7 (0–14)	15
Chicken and other poultry	29 (0–58)	62
Eggs	13 (0–25)	19
Fish§	28 (0–100)	40
Legumes		
Dry beans, lentils, and peas*	50 (0–100)	172
Soy foods	25 (0–50)	112
Peanuts	25 (0–75)	142
Tree nuts	25	149
Added fats		
Palm oil¶	6·8 (0–6·8)	60
Unsaturated oils	40 (20–80)	354
Dairy fats (included in milk)	0	0
Lard or tallow||	5 (0–5)	36
Added sugars		
All sweeteners	31 (0–31)	120

For an individual, an optimal energy intake to maintain a healthy weight will depend on body size and level of physical activity. Processing of foods such as partial hydrogenation of oils, refining of grains, and addition of salt and preservatives can substantially affect health but is not addressed in this table. *Wheat, rice, dry beans, and lentils are dry, raw. †Mix and amount of grains can vary to maintain isocaloric intake. ‡Beef and lamb are exchangeable with pork and vice versa. Chicken and other poultry is exchangeable with eggs, fish, or plant protein sources. Legumes, peanuts, tree nuts, seeds, and soy are interchangeable. §Seafood consist of fish and shellfish (e.g., mussels and shrimps) and originate from both capture and from farming. Although seafood is a highly diverse group that contains both animals and plants, the focus of this report is solely on animals. ¶Unsaturated oils are 20% each of olive, soybean, rapeseed, sunflower, and peanut oil. ||Some lard or tallow are optional in instances when pigs or cattle are consumed.[1]

© 2023 Ye Y et al. *JAMA Network Open.*

eTable 2. Construction of the Planetary Health Diet Score in the Singapore Chinese Health Study

Type	Dietary components	Original intake (range) of the PHD components, for an intake of 2500 kcal/day	Food items based on the available foods in the FFQ in the SCHS	Criteria for score distribution based on PHD (g/2500 kcal)				
				Minimum score (0 points)	Proportional score (0-10 points)	Maximum score (10 points)	Proportional score (10-0 points)	Minimum score (0 points)
	**Total grains**							
M	1. Rice, wheat, corn, and other	232 (total grains 0-60% of energy) g/day	Rice, noodles, breads, cereals			≤232	232-464	≥464
	**Tubers of starchy vegetables**							
O	2. Potatoes and cassava	50 (0-100) g/day	Potatoes	0	0-50	50	50-100	≥100
	**Vegetables**							
A	3. All vegetables (dark green vegetables, red and orange vegetables, other vegetables)	300 (200-600) g/day	Green vegetables (light green and dark green), cruciferous vegetables, yellow-orange vegetables, orange vegetables, and tomato products	0	0-300	≥300		
	**Fruit**							
A	4. All fruits	200 (100-300) g/day	Citrus fruits, other fruits	0	0-200	≥200		
	**Dairy foods**							
O	5. Whole milk or derivative equivalents	250 (0-500) g/day	Milk including powder	0	0-250	250	250-500	≥500
	**Protein sources**							
M	6. Beef, lamb, pork	14 (0-28) g/day	Red meat, processed meat			≤14	14-28	≥28
O	7. Chicken and other poultry	29 (0-58) g/day	Poultry	0	0-29	29	29-58	≥58
O	8. Eggs	13 (0-25) g/day	Eggs	0	0-13	13	13-25	≥25
A	9. Fish	28 (0-100) g/day	Fresh fish, shellfish	0	0-28	≥28		
	**Legumes**							
A	10. Dry beans, lentils, peas, soy foods	75 (0-150) g/day	Non-soy legumes, tofu products, soybean drink	0	0-75	≥75		

© 2023 Ye Y et al. *JAMA Network Open.*

A	11. Peanuts or tree nuts	50 (0-75) g/day	Nuts, seeds	0	0-50	≥50		
	**Added fats**							
M	12. Palm oil, dairy fats (included in milk), lard or tallow	11.8 (0-11.8) g/day	SFAs			0	0-11.8	≥11.8
A	13. Unsaturated oils	40 (20-80) g/day	MUFAs, PUFAs	0	0-40	≥40		
	**Added sugar**							
M	14. All sweeteners	31 (0-31) g/day	Added sugar			0	0-31	≥31

Abbreviations: A, adequacy components; FFQ, food frequency questionnaire; M, moderation components; MUFA, monounsaturated fatty acid; O, optimum components; PHD, planetary health diet; PUFA, polyunsaturated fatty acid; SCHS, Singapore Chinese Health Study; SFA, saturated fatty acid.

© 2023 Ye Y et al. *JAMA Network Open.*

eTable 3. Median Intake and Complete Adherence (%) of Dietary Components by Quintiles of the Planetary Health Diet Score from the Singapore Chinese Health Study

Variables	All	Quintiles of planetary health diet score				
		Q1	Q2	Q3	Q4	Q5
Median intake of dietary components, g/d						
Vegetables	147.25 (106.83-199.25)	106.56 (76.29-143.31)	129.33 (97.88-169.16)	145.74 (110.84-188.76)	165.57 (126.17-212.69)	204.30 (155.64-265.53)
Fruits	280.36 (154.87-440.84)	112.81 (42.40-241.15)	247.12 (138.65-396.28)	292.54 (187.33-444.27)	329.13 (220.11-479.83)	388.85 (265.37-552.14)
Nuts	2.34 (0.70-4.82)	1.57 (0.30-3.62)	2.21 (0.68-4.55)	2.45 (0.77-4.85)	2.65 (0.93-5.35)	2.85 (1.01-5.92)
Legumes	27.45 (17.12-40.96)	18.30 (10.71-27.91)	24.03 (15.70-35.02)	27.92 (18.30-39.84)	31.56 (20.71-44.74)	38.30 (25.98-54.73)
Unsaturated fats	37.52 (31.50-43.67)	33.11 (26.86-39.91)	35.99 (30.21-42.13)	37.52 (31.96-43.35)	38.73 (33.41-44.57)	41.19 (36.05-46.98)
Fish	80.62 (55.41-109.66)	72.15 (45.87-101.19)	79.24 (54.74-107.21)	81.08 (56.96-109.18)	83.98 (58.98-112.99)	86.37 (60.85-116.82)
Total grains	856.93 (710.20-1017.04)	961.09 (798.06-1127.13)	887.80 (738.58-1047.89)	858.13 (715.42-1008.96)	825.24 (691.98-969.32)	771.52 (634.69-914.11)
Red meat	43.50 (27.43-62.85)	46.79 (32.00-67.09)	46.39 (31.14-65.52)	44.95 (29.48-63.57)	42.95 (26.15-61.90)	35.28 (18.04-55.27)
Saturated fats	24.36 (19.62-29.40)	22.71 (17.88-28.03)	23.96 (19.23-28.68)	24.58 (20.03-29.29)	24.89 (20.32-29.93)	25.78 (20.72-30.75)
Added sugar	17.53 (10.03-27.38)	24.31 (15.21-34.50)	19.62 (11.48-29.42)	17.38 (10.20-26.59)	15.23 (8.64-23.48)	13.10 (7.02-20.40)
Potatoes	5.56 (2.11-10.47)	3.35 (0.49-7.08)	4.56 (1.68-8.56)	5.50 (2.25-10.07)	6.59 (2.88-11.59)	8.95 (4.04-16.34)
Dairy foods	35.69 (8.12-117.45)	18.89 (3.97-54.86)	27.95 (6.19-71.72)	34.68 (8.98-96.82)	42.49 (11.77-151.67)	82.84 (25.12-256.89)
Poultry	26.89 (14.60-43.09)	24.71 (9.60-53.73)	27.90 (13.64-49.17)	27.67 (15.57-44.13)	27.30 (16.94-39.38)	26.28 (17.27-34.85)
Eggs	13.64 (7.02-25.11)	19.66 (5.38-33.10)	16.05 (6.36-28.84)	13.90 (7.16-24.97)	12.73 (7.42-20.36)	12.41 (8.26-16.87)
Complete adherence (%)						
Vegetables	5.594	0.163	0.335	0.715	1.268	3.113
Fruits	66.027	6.062	12.038	14.382	15.933	17.613
Nuts	0.135	0.002	0.012	0.009	0.030	0.082
Legumes	3.672	0.200	0.350	0.545	0.853	1.724
Unsaturated fats	39.255	4.925	6.572	7.725	8.769	11.265
Fish	93.740	17.678	18.702	18.958	19.172	19.230
Total grains	0.072	0.005	0.011	0.000	0.004	0.053
Red meat	8.536	0.965	1.067	1.375	1.831	3.297
Saturated fats	0.000	0.000	0.000	0.000	0.000	0.000

© 2023 Ye Y et al. *JAMA Network Open.*

Added sugar	0.037	0.005	0.004	0.007	0.009	0.012
Potatoes	0.000	0.000	0.000	0.000	0.000	0.000
Dairy foods	0.000	0.000	0.000	0.000	0.000	0.000
Poultry	0.000	0.000	0.000	0.000	0.000	0.000
Eggs	0.000	0.000	0.000	0.000	0.000	0.000

Values are median (interquartile range) and percentage of each dietary component that completely adhered to (10 points) each quintile of the planetary health diet score.

© 2023 Ye Y et al. *JAMA Network Open.*

eTable 4. GHG Emissions (kg CO_2e) Across Quintiles of the Planetary Health Diet Score from Different Dietary Components

Dietary components	Quintiles of planetary health diet score					*P* trend
	Q1	Q2	Q3	Q4	Q5	
Total grains	1.65 (0.40)	1.53 (0.38)	1.48 (0.36)	1.42 (0.35)	1.32 (0.36)	<.001
Vegetables	0.07 (0.03)	0.09 (0.04)	0.10 (0.04)	0.11 (0.05)	0.14 (0.06)	<.001
Fruits	0.02 (0.02)	0.03 (0.02)	0.03 (0.02)	0.04 (0.02)	0.04 (0.03)	<.001
Nuts	0.002 (0.002)	0.002 (0.003)	0.002 (0.003)	0.002 (0.003)	0.003 (0.004)	<.001
Potatoes	0.001 (0.001)	0.001 (0.001)	0.001 (0.001)	0.002 (0.002)	0.002 (0.002)	<.001
Legumes	0.01 (0.01)	0.02 (0.01)	0.02 (0.01)	0.02 (0.01)	0.02 (0.01)	<.001
Fish	0.27 (0.15)	0.29 (0.14)	0.30 (0.15)	0.31 (0.15)	0.32 (0.15)	<.001
Eggs	0.05 (0.06)	0.05 (0.05)	0.04 (0.04)	0.04 (0.03)	0.03 (0.03)	<.001
Dairy	0.09 (0.21)	0.11 (0.21)	0.13 (0.21)	0.14 (0.21)	0.18 (0.21)	<.001
Poultry	0.08 (0.07)	0.08 (0.07)	0.08 (0.06)	0.08 (0.05)	0.07 (0.04)	<.001
Unsaturated fats	0.14 (0.04)	0.15 (0.04)	0.16 (0.04)	0.16 (0.04)	0.17 (0.04)	<.001
Saturated fats	0.10 (0.03)	0.10 (0.03)	0.10 (0.03)	0.10 (0.03)	0.11 (0.03)	<.001
Red meat	0.27 (0.15)	0.27 (0.15)	0.26 (0.14)	0.24 (0.14)	0.21 (0.14)	<.001
Added sugar	0.010 (0.006)	0.008 (0.005)	0.007 (0.005)	0.007 (0.005)	0.006 (0.004)	<.001

Abbreviations: CO_2e, CO_2 equivalent; GHG, greenhouse gas emissions.
Values are presented as mean (standard deviation).
P trends were assessed by using linear regression models with the median value of the quintiles of planetary health diet score as a continuous variable after adjusting for total energy intake.

© 2023 Ye Y et al. *JAMA Network Open.*

eTable 5. TWF (m^3) Across Quintiles of the Planetary Health Diet Score from Different Dietary Components

Dietary components	Quintiles of planetary health diet score					*P* trend
	Q1	Q2	Q3	Q4	Q5	
Total grains	1.03 (0.25)	0.95 (0.24)	0.92 (0.23)	0.89 (0.22)	0.83 (0.22)	<.001
Vegetables	0.02 (0.01)	0.03 (0.01)	0.03 (0.01)	0.03 (0.01)	0.04 (0.02)	<.001
Fruits	0.12 (0.13)	0.20 (0.15)	0.23 (0.15)	0.25 (0.15)	0.30 (0.17)	<.001
Nuts	0.002 (0.003)	0.003 (0.003)	0.003 (0.004)	0.003 (0.004)	0.004 (0.006)	<.001
Potatoes	0.002 (0.002)	0.002 (0.002)	0.002 (0.003)	0.003 (0.003)	0.004 (0.004)	<.001
Legumes	0.06 (0.04)	0.08 (0.05)	0.09 (0.05)	0.10 (0.06)	0.12 (0.07)	<.001
Fish	0.06 (0.03)	0.06 (0.03)	0.06 (0.03)	0.07 (0.03)	0.07 (0.03)	<.001
Eggs	0.05 (0.05)	0.04 (0.04)	0.04 (0.04)	0.04 (0.03)	0.03 (0.03)	<.001
Dairy	0.09 (0.20)	0.11 (0.20)	0.12 (0.20)	0.14 (0.20)	0.17 (0.20)	<.001
Poultry	0.05 (0.05)	0.05 (0.04)	0.05 (0.04)	0.05 (0.03)	0.04 (0.03)	<.001
Unsaturated fats	0.20 (0.06)	0.22 (0.05)	0.23 (0.05)	0.23 (0.05)	0.25 (0.05)	<.001
Saturated fats	0.44 (0.14)	0.45 (0.13)	0.47 (0.13)	0.47 (0.13)	0.49 (0.13)	<.001
Red meat	0.28 (0.16)	0.28 (0.15)	0.27 (0.15)	0.25 (0.15)	0.22 (0.15)	<.001
Added sugar	0.04 (0.02)	0.03 (0.02)	0.03 (0.02)	0.03 (0.02)	0.02 (0.02)	<.001

Abbreviation: TWF, total water footprint.
Values are presented as mean (standard deviation).
P trends were assessed by using linear regression models with the median value of the quintiles of planetary health diet score as a continuous variable after adjusting for total energy intake.

© 2023 Ye Y et al. *JAMA Network Open.*

eTable 6. Land Use (m^2) Across Quintiles of the Planetary Health Diet Score from Different Dietary Components

Dietary components	Quintiles of planetary health diet score					*P* trend
	Q1	Q2	Q3	Q4	Q5	
Total grains	1.17 (0.29)	1.08 (0.27)	1.04 (0.26)	1.01 (0.25)	0.94 (0.25)	<.001
Vegetables	0.06 (0.03)	0.07 (0.03)	0.08 (0.03)	0.09 (0.04)	0.11 (0.05)	<.001
Fruits	0.18 (0.20)	0.30 (0.23)	0.35 (0.23)	0.38 (0.23)	0.45 (0.26)	<.001
Nuts	0.005 (0.007)	0.006 (0.008)	0.007 (0.009)	0.008 (0.010)	0.009 (0.013)	<.001
Potatoes	0.003 (0.005)	0.004 (0.005)	0.005 (0.005)	0.006 (0.006)	0.008 (0.008)	<.001
Legumes	0.12 (0.09)	0.16 (0.10)	0.18 (0.11)	0.20 (0.12)	0.24 (0.14)	<.001
Fish	0.16 (0.09)	0.18 (0.09)	0.18 (0.09)	0.19 (0.09)	0.20 (0.09)	<.001
Eggs	0.21 (0.22)	0.18 (0.18)	0.16 (0.15)	0.15 (0.14)	0.13 (0.11)	<.001
Dairy	0.28 (0.61)	0.34 (0.63)	0.38 (0.63)	0.43 (0.64)	0.55 (0.62)	<.001
Poultry	0.29 (0.25)	0.29 (0.22)	0.27 (0.20)	0.26 (0.18)	0.23 (0.15)	<.001
Unsaturated fats	0.08 (0.02)	0.08 (0.02)	0.09 (0.02)	0.09 (0.02)	0.09 (0.02)	<.001
Saturated fats	0.10 (0.03)	0.10 (0.03)	0.10 (0.03)	0.10 (0.03)	0.11 (0.03)	<.001
Red meat	0.38 (0.21)	0.37 (0.20)	0.35 (0.20)	0.33 (0.19)	0.29 (0.19)	<.001
Added sugar	0.0005 (0.0003)	0.0004 (0.0003)	0.0004 (0.0003)	0.0004 (0.0003)	0.0003 (0.0002)	<.001

Values are presented as mean (standard deviation).
P trends were assessed by using linear regression models with the median value of the quintiles of planetary health diet score as a continuous variable after adjusting for total energy intake.

© 2023 Ye Y et al. *JAMA Network Open.*

eTable 7. Hazard Ratios and 95% Confidence Intervals for the Association Between Planetary Health Diet Score and Subtypes of Cardiovascular Disease Mortality and Respiratory Disease Mortality in the Singapore Chinese Health Study (n=57078)

	Quintiles of planetary health diet score					*P* trend[a]
	Q1	Q2	Q3	Q4	Q5	
**CVD mortality**						
**Ischemic heart disease**						
Events	1012	752	743	713	610	NA
Model 1	1.00 (Reference)	0.77 (0.70-0.84)	0.78 (0.71-0.86)	0.79 (0.71-0.87)	0.73 (0.66-0.81)	<.001
Model 2	1.00 (Reference)	0.78 (0.71-0.86)	0.79 (0.72-0.87)	0.79 (0.72-0.88)	0.73 (0.66-0.81)	<.001
**Stroke (all types)**						
Events	464	379	386	319	318	NA
Model 1	1.00 (Reference)	0.85 (0.75-0.98)	0.90 (0.79-1.03)	0.77 (0.67-0.89)	0.83 (0.72-0.96)	.003
Model 2	1.00 (Reference)	0.90 (0.78-1.03)	0.94 (0.82-1.08)	0.81 (0.70-0.94)	0.89 (0.77-1.03)	.04
**Hemorrhage stroke**						
Events	114	106	84	76	89	NA
Model 1	1.00 (Reference)	0.95 (0.73-1.23)	0.77 (0.58-1.02)	0.71 (0.53-0.95)	0.88 (0.67-1.17)	.11
Model 2	1.00 (Reference)	0.99 (0.76-1.29)	0.81 (0.61-1.08)	0.76 (0.57-1.02)	0.97 (0.73-1.30)	.36
**Ischemic/unspecified stroke**						
Events	350	273	302	242	229	NA
Model 1	1.00 (Reference)	0.82 (0.70-0.97)	0.95 (0.81-1.10)	0.79 (0.67-0.93)	0.81 (0.68-0.96)	.01
Model 2	1.00 (Reference)	0.87 (0.74-1.02)	0.98 (0.84-1.15)	0.83 (0.70-0.98)	0.86 (0.72-1.02)	.07
**Respiratory disease mortality**						
**Pneumonia**						
Events	964	822	770	758	630	NA
Model 1	1.00 (Reference)	0.85 (0.78-0.94)	0.83 (0.76-0.92)	0.86 (0.78-0.95)	0.78 (0.70-0.86)	<.001
Model 2	1.00 (Reference)	0.89 (0.81-0.98)	0.88 (0.80-0.97)	0.92 (0.83-1.01)	0.83 (0.75-0.92)	.002
**Chronic obstructive pulmonary disease**						
Events	287	200	138	102	80	NA
Model 1	1.00 (Reference)	0.75 (0.63-0.90)	0.56 (0.45-0.68)	0.44 (0.35-0.56)	0.39 (0.31-0.51)	<.001
Model 2	1.00 (Reference)	0.95 (0.79-1.14)	0.80 (0.65-0.98)	0.67 (0.54-0.85)	0.65 (0.50-0.84)	<.001

Abbreviations: CVD, cardiovascular disease; NA, not applicable.

[a] Linear trends were assessed by treating the median values of the quintiles of planetary health diet score as a continuous variable.
Model 1: Adjusted for age, sex, and energy intake (kcal/d).
Model 2: Additionally adjusted for dialect group (Cantonese or Hokkien), educational level (no formal education, primary school, or secondary school or higher), body mass index (kg/m^2), smoking status (never, former, or current), alcohol frequency (none, monthly, weekly, or daily), physical activity (<0.5 h/wk, 0.5-3.9 h/wk, or ≥4 h/wk), sleep duration (<6 h/d, 6-8 h/d, or >8 h/d), and self-reported history of physician-diagnosed hypertension and diabetes.

© 2023 Ye Y et al. *JAMA Network Open.*

eTable 8. Subgroup Analyses of the Association Between Planetary Health Diet Score and All-Cause Mortality in the Singapore Chinese Health Study

Groups	Case/N	Quintiles of planetary health diet score					*P* for interaction[a]
		Q1	Q2	Q3	Q4	Q5	
**All-cause mortality**							
Age (years)							.96
<55	4900/27085	1.00 (Reference)	0.89 (0.82-0.97)	0.91 (0.83-0.99)	0.90 (0.82-0.98)	0.87 (0.80-0.96)	
≥55	17699/29993	1.00 (Reference)	0.91 (0.87-0.95)	0.88 (0.84-0.92)	0.87 (0.83-0.92)	0.85 (0.81-0.89)	
Sex (sex-specific quintiles)[b]							.04
Men	12008/25120	1.00 (Reference)	0.94 (0.89-0.99)	0.88 (0.83-0.93)	0.88 (0.83-0.93)	0.86 (0.81-0.91)	
Women	10591/31958	1.00 (Reference)	0.85 (0.80-0.90)	0.89 (0.84-0.95)	0.86 (0.81-0.91)	0.83 (0.78-0.88)	
BMI (kg/m^2)							.82
<23	10597/27569	1.00 (Reference)	0.90 (0.86-0.96)	0.91 (0.86-0.97)	0.90 (0.85-0.95)	0.86 (0.81-0.92)	
≥23	12002/29509	1.00 (Reference)	0.90 (0.86-0.96)	0.87 (0.82-0.92)	0.86 (0.81-0.91)	0.84 (0.79-0.89)	
Smoking status							.01
Never smoker	12912/40044	1.00 (Reference)	0.90 (0.86-0.96)	0.91 (0.86-0.96)	0.88 (0.84-0.93)	0.86 (0.81-0.91)	
Ever smoker	9687/17034	1.00 (Reference)	0.89 (0.84-0.94)	0.84 (0.79-0.89)	0.84 (0.79-0.90)	0.80 (0.75-0.86)	

Abbreviations: BMI, body mass index; CVD, cardiovascular disease.

[a] The Likelihood ratio test was used for testing interactions.

All models were adjusted for age, sex, energy intake, dialect group (Cantonese or Hokkien), educational level (no formal education, primary school, or secondary school or higher), body mass index (kg/m^2), smoking status (never, former, or current), alcohol frequency (none, monthly, weekly, or daily), physical activity (<0.5 h/wk, 0.5-3.9 h/wk, or ≥4 h/wk), sleep duration (<6 h/d, 6-8 h/d, or >8 h/d), and self-reported history of physician-diagnosed hypertension and diabetes except for the grouping variables.

[b] Sex-specific quintiles of the planetary health diet score were used to perform the analysis.

© 2023 Ye Y et al. *JAMA Network Open.*

eTable 9. Sensitivity Analyses of Multivariable-adjusted Association of Planetary Health Diet Score with All-Cause and Cause-Specific Mortality in the Singapore Chinese Health Study

	Quintiles of planetary health diet score					*P* trend[a]
	Q1	Q2	Q3	Q4	Q5	
Sensitivity 1: Excluding participants with history of hypertension or diabetes at baseline (n=42057)[b]						
All-cause mortality	1.00 (Reference)	0.90 (0.86-0.94)	0.89 (0.85-0.93)	0.89 (0.84-0.93)	0.85 (0.81-0.90)	<.001
CVD mortality	1.00 (Reference)	0.82 (0.75-0.90)	0.83 (0.75-0.91)	0.80 (0.72-0.88)	0.76 (0.68-0.84)	<.001
Cancer mortality	1.00 (Reference)	0.95 (0.88-1.03)	0.92 (0.85-1.00)	0.96 (0.88-1.05)	0.95 (0.87-1.04)	.25
Respiratory diseases mortality	1.00 (Reference)	0.88 (0.80-0.96)	0.88 (0.80-0.98)	0.85 (0.76-0.94)	0.78 (0.69-0.87)	<.001
Sensitivity 2: Excluding participants who died within 5 years from recruitment (n=54627)[c]						
All-cause mortality	1.00 (Reference)	0.88 (0.85-0.92)	0.88 (0.84-0.91)	0.88 (0.84-0.92)	0.85 (0.81-0.89)	<.001
CVD mortality	1.00 (Reference)	0.82 (0.76-0.89)	0.84 (0.77-0.90)	0.80 (0.74-0.86)	0.78 (0.72-0.85)	<.001
Cancer mortality	1.00 (Reference)	0.92 (0.85-0.99)	0.91 (0.85-0.99)	0.95 (0.88-1.02)	0.92 (0.85-0.99)	.07
Respiratory diseases mortality	1.00 (Reference)	0.89 (0.82-0.97)	0.87 (0.80-0.95)	0.89 (0.81-0.97)	0.82 (0.74-0.90)	<.001

Abbreviation: CVD, cardiovascular disease.

[a] Linear trends were assessed by treating the median values of the quintiles of planetary health diet score as a continuous variable.

[b] Adjusted for age, sex, energy intake (kcal/d), dialect group (Cantonese or Hokkien), educational level (no formal education, primary school, or secondary school or higher), body mass index (kg/m^2), smoking status (never, former, or current), alcohol frequency (none, monthly, weekly, or daily), physical activity (<0.5 h/wk, 0.5-3.9 h/wk, or ≥4 h/wk), and sleep duration (<6 h/d, 6-8 h/d, or >8 h/d).

[c] Additionally adjusted for self-reported history of physician-diagnosed hypertension and diabetes at baseline.

© 2023 Ye Y et al. *JAMA Network Open.*

eTable 10. Multivariable-adjusted Association Between Previously Established Planetary Health Diet Score and All-Cause and Cause-Specific Mortality in the Singapore Chinese Health Study[a]

	Tertiles of planetary health diet score			*P* trend[b]
	T1	T2	T3	
Participants	30593	17615	8870	NA
Planetary healthy diet score, median (interquartile range)	8 (7-8)	9 (9-9)	10 (10-10)	NA
All-cause mortality	1.00 (Reference)	0.94 (0.91-0.97)	0.88 (0.85-0.92)	<.001
CVD mortality	1.00 (Reference)	0.96 (0.91-1.01)	0.87 (0.81-0.94)	<.001
Cancer mortality	1.00 (Reference)	0.94 (0.89-0.99)	0.90 (0.84-0.96)	<.001
Respiratory diseases mortality	1.00 (Reference)	0.91 (0.85-0.97)	0.86 (0.79-0.94)	<.001

Abbreviations: CVD, cardiovascular disease; NA, not applicable.

[a] The score (0–14 points) was developed by Knuppel[2] et al. and reflects recommended consumption amounts in the planetary health diet.

[b] Linear trends were assessed by treating the median values of the tertiles of planetary health diet score as a continuous variable.

Models were adjusted for age, sex, energy intake, dialect group (Cantonese or Hokkien), educational level (no formal education, primary school, or secondary school or higher), body mass index (kg/m^2), smoking status (never, former, or current), alcohol frequency (none, monthly, weekly, or daily), physical activity (<0.5 h/wk, 0.5-3.9 h/wk, or ≥4 h/wk), sleep duration (<6 h/d, 6-8 h/d, or >8 h/d), and self-reported history of physician-diagnosed hypertension and diabetes at baseline.

© 2023 Ye Y et al. *JAMA Network Open.*

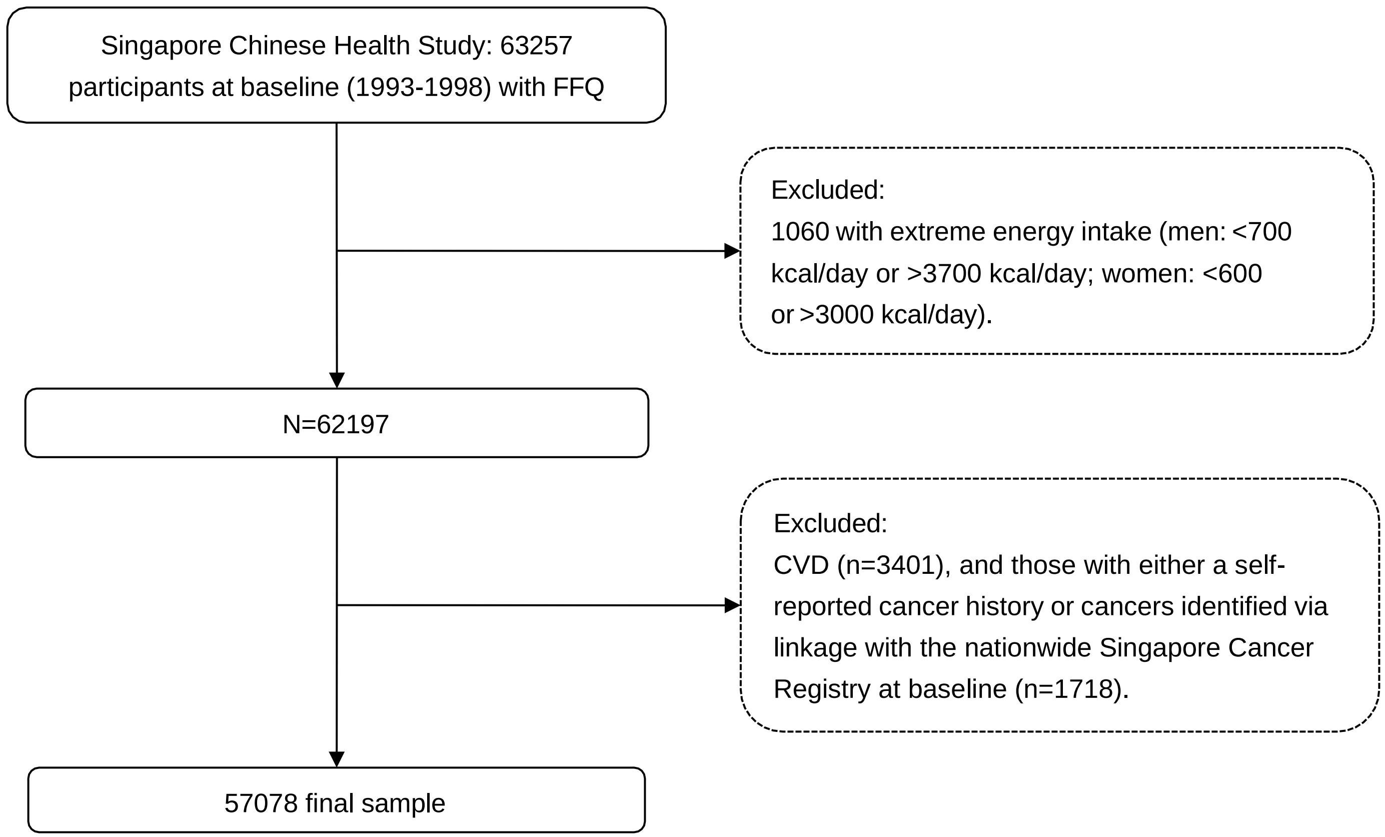

eFigure 1. Flow Chart of Final Sample from the Singapore Chinese Health Study

Abbreviations: CVD, cardiovascular disease; FFQ, food frequency questionnaire.

© 2023 Ye Y et al. *JAMA Network Open.*

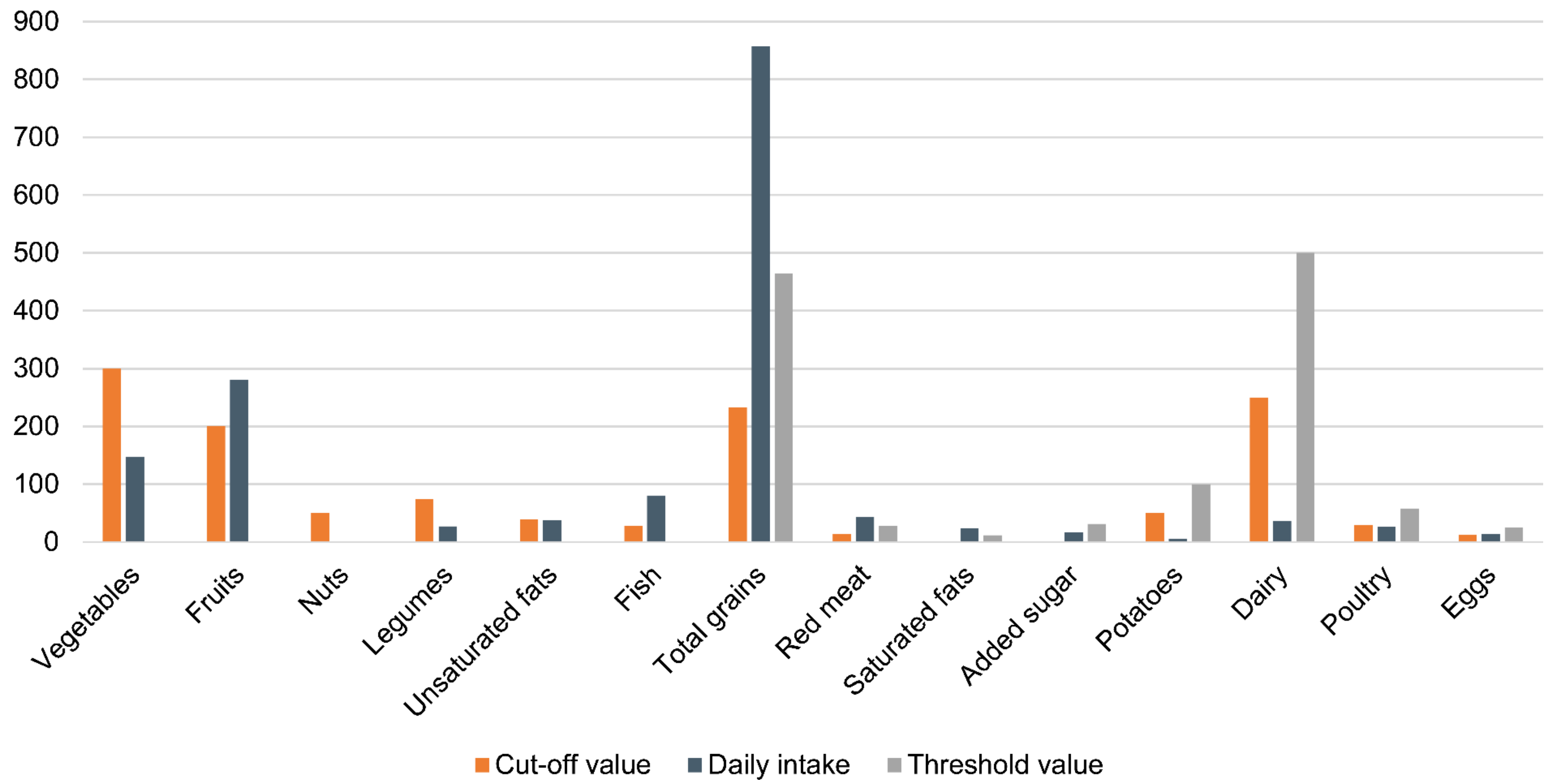

eFigure 2. Comparison of the Recommended Range of the Planetary Health Diet and Median Intake of the Participants in the Singapore Chinese Health Study

© 2023 Ye Y et al. *JAMA Network Open.*

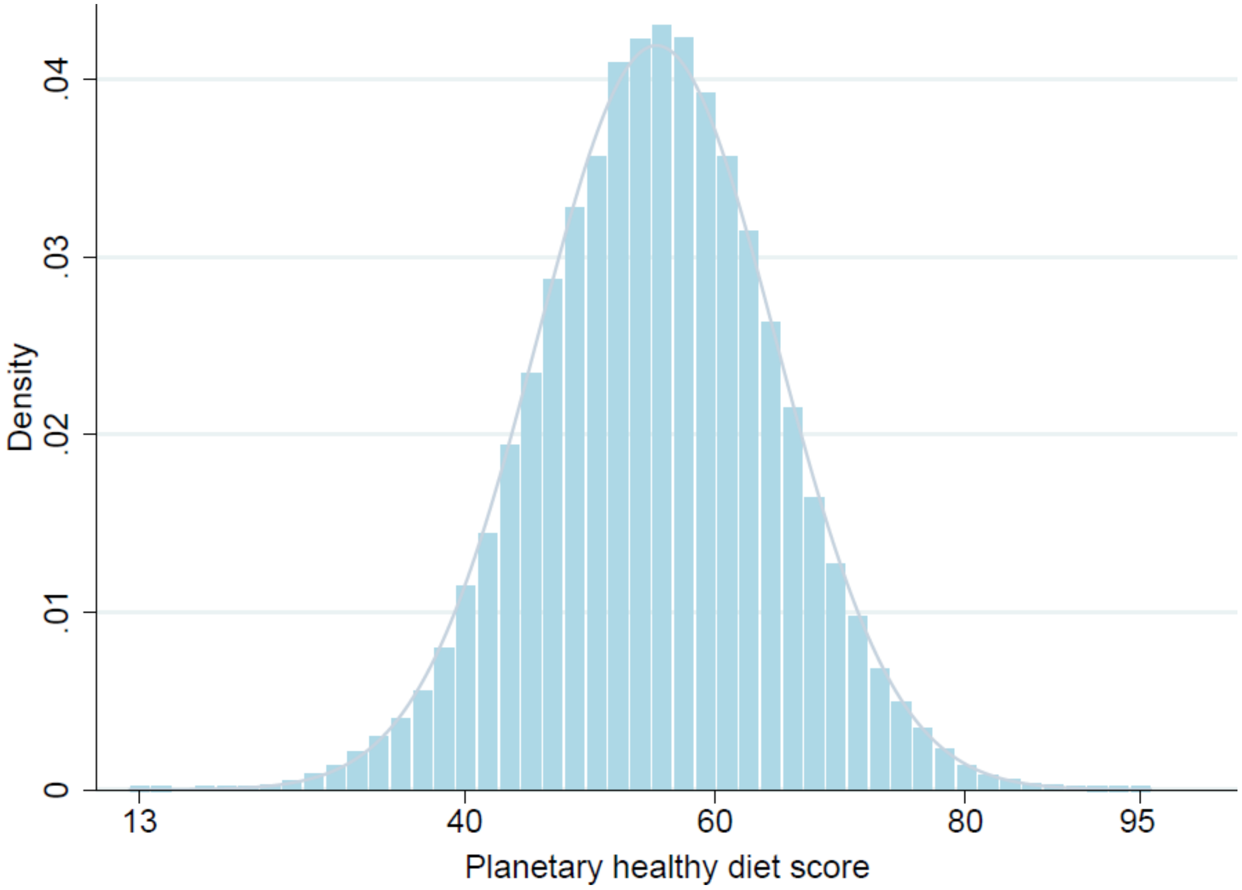

eFigure 3. Distribution of Planetary Health Diet Score in the Singapore Chinese Health Study

© 2023 Ye Y et al. *JAMA Network Open.*

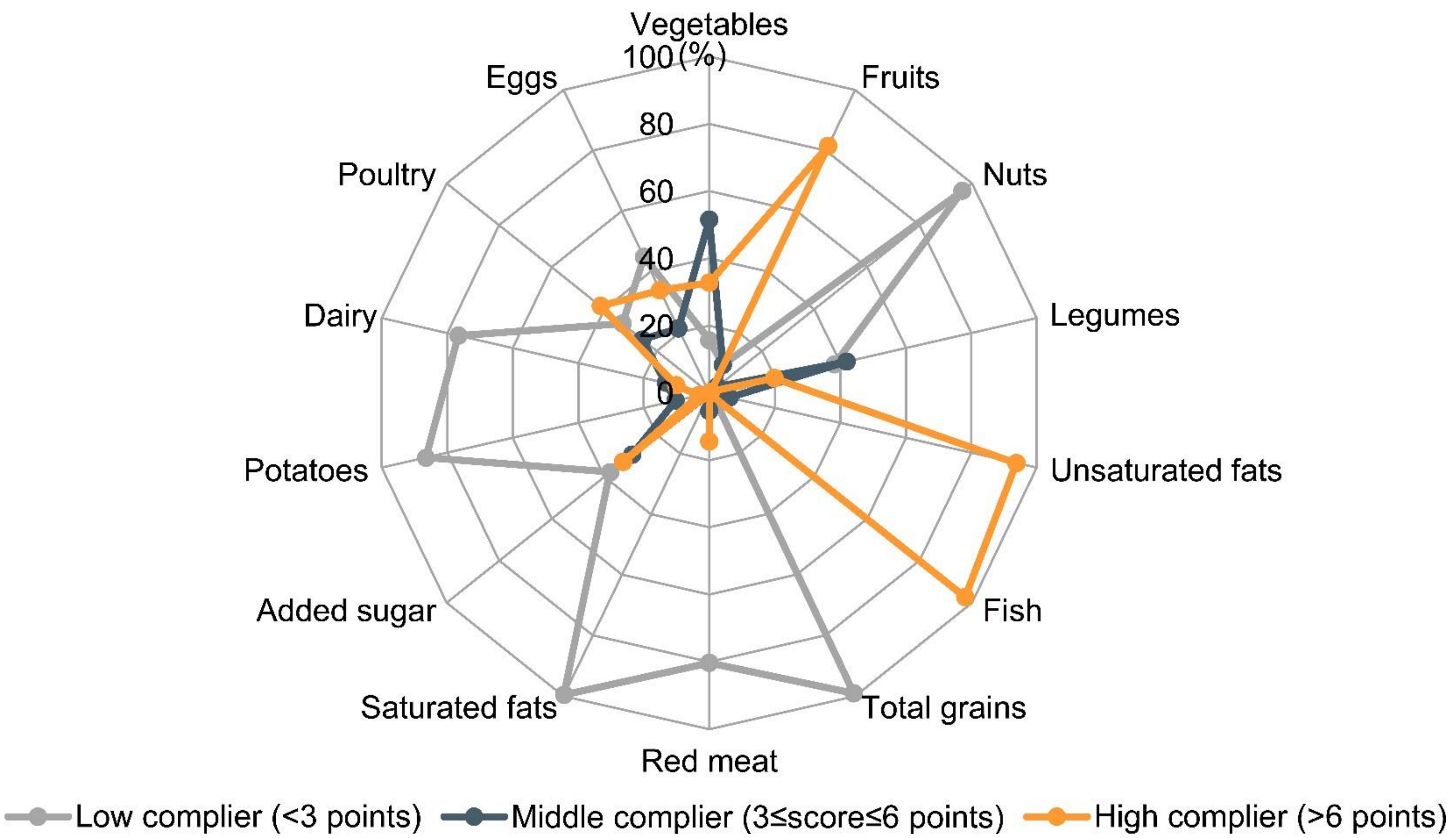

eFigure 4. Compliance with Planetary Health Diet According to Score for Each Dietary Component

The radar chart plots the values of each dietary component along a separate axis that starts in the center of the chart (0% compliance) and ends at the outer ring (100% compliance). The values are the percentage of participants who adhered to each dietary recommendation.

© 2023 Ye Y et al. *JAMA Network Open.*

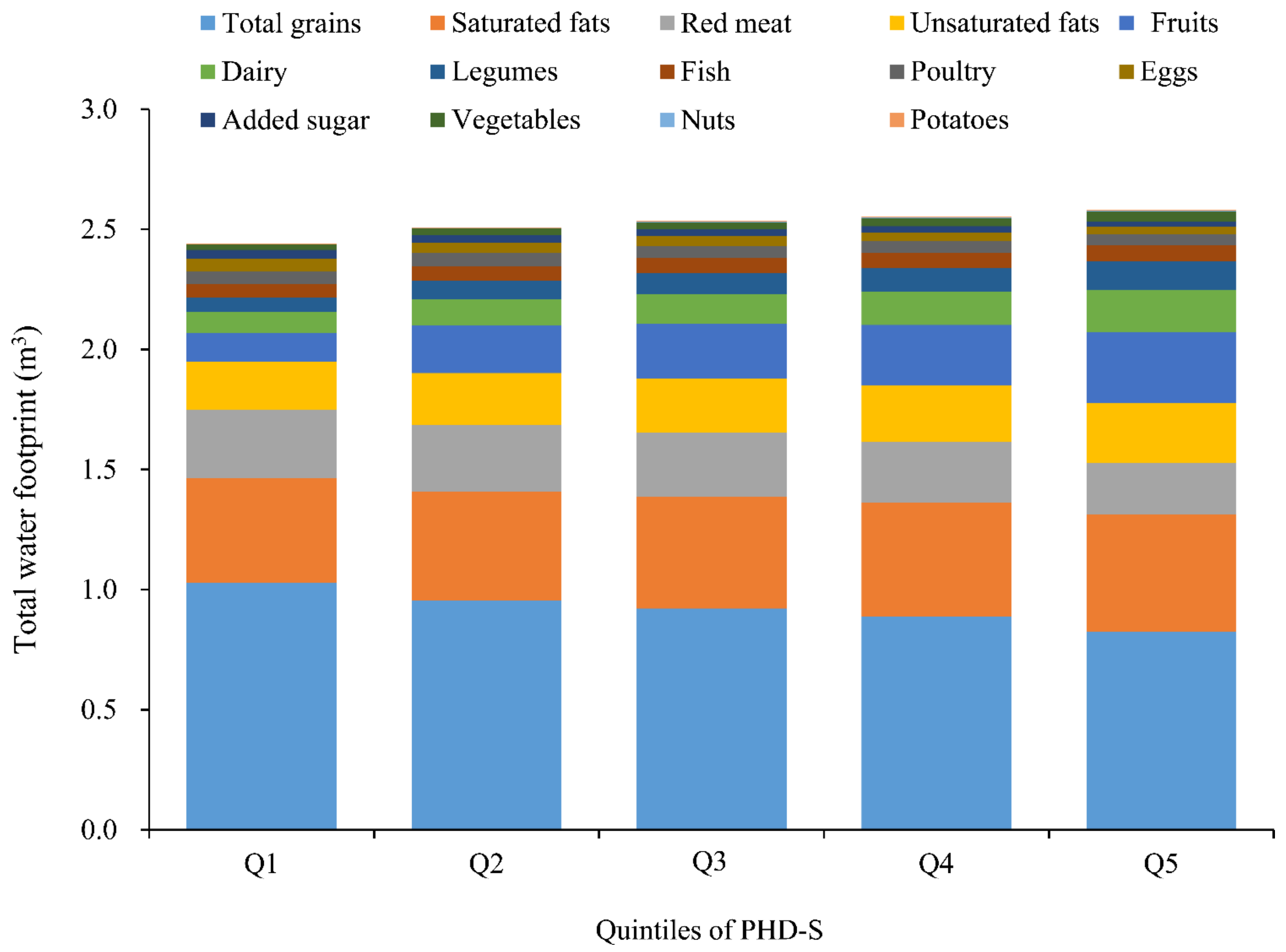

eFigure 5. Total Water Footprint Across Quintiles of the Planetary Health Diet Score from Different Dietary Components

Abbreviation: PHD-S, planetary healthy diet score.

© 2023 Ye Y et al. *JAMA Network Open.*

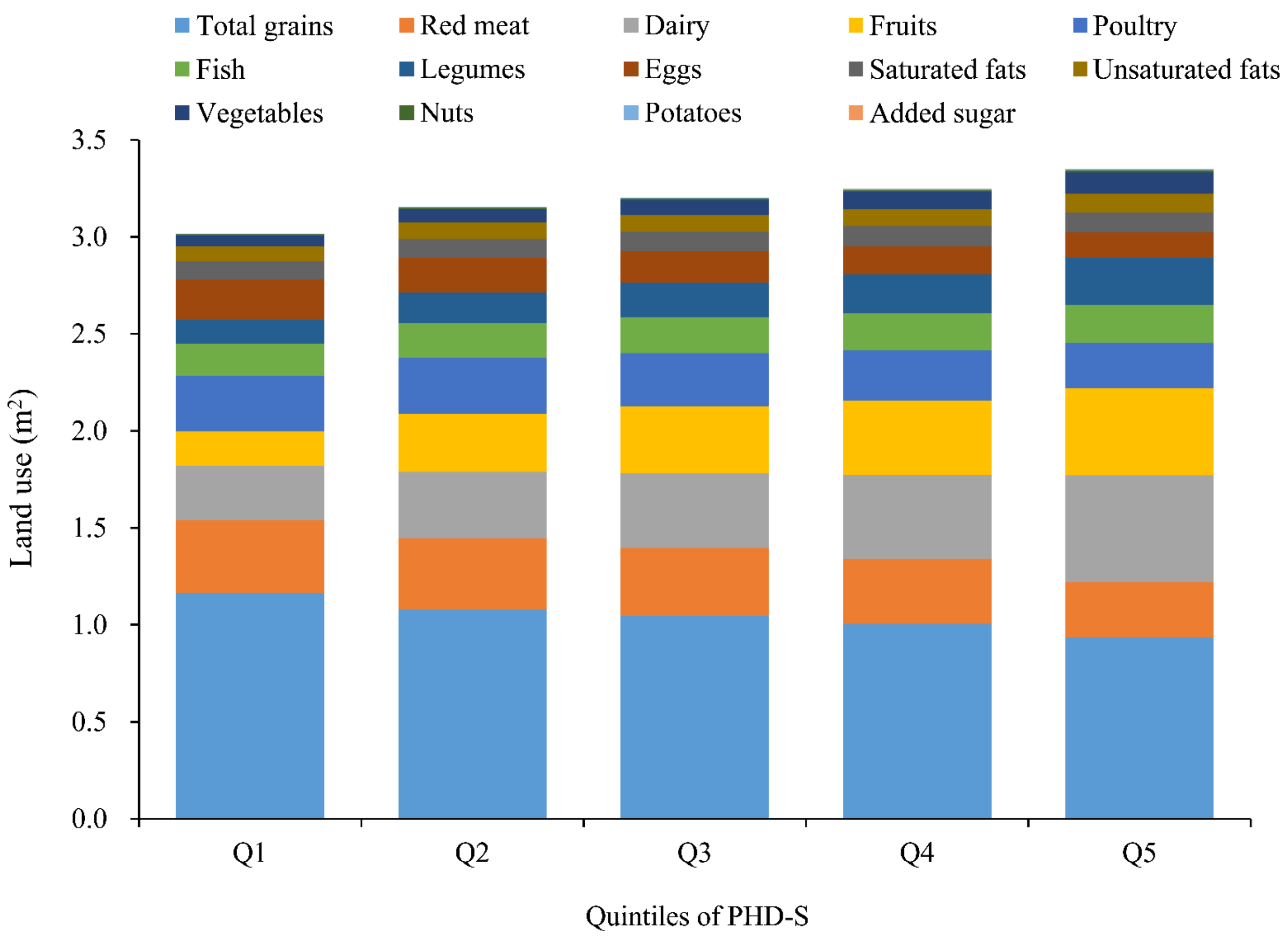

eFigure 6. Land Use Across Quintiles of the Planetary Health Diet Score from Different Dietary Components

Abbreviation: PHD-S, planetary health diet score.

© 2023 Ye Y et al. *JAMA Network Open.*

eReferences

1. Willett W, Rockström J, Loken B, et al. Food in the Anthropocene: the EAT-Lancet Commission on healthy diets from sustainable food systems. *Lancet.* 2019;393(10170):447-492. doi:10.1016/S0140-6736(18)31788-4
2. Knuppel A, Papier K, Key TJ, Travis RC. EAT-Lancet score and major health outcomes: the EPIC-Oxford study. *Lancet.* 2019;394(10194):213-214. doi:10.1016/S0140-6736(19)31236-X

© 2023 Ye Y et al. *JAMA Network Open.*
