# Supplementary material for: Adherence to a Planetary Health Diet, Environmental Impacts, and Mortality in Chinese Adults
Source: JAMA Netw Open. 2023 Oct 24;6(10):e2339468. doi: 10.1001/jamanetworkopen.2023.39468 (PMC10599124; doi:10.1001/jamanetworkopen.2023.39468)
Supplement: Supplement 2. — Data Sharing Statement [file jamanetwopen-e2339468-s002.pdf]

Data Sharing Statement

Ye. Adherence to a Planetary Healthy Diet, Environmental Impacts, and Mortality in Chinese Adults. *JAMA Netw Open*. Published October 24, 2023.
doi:10.1001/jamanetworkopen.2023.39468

Data
Data available: Yes
Data types: Data dictionary
How to access data: Data will be made available with reasonable request or data analysis proposal to the corresponding authors upon the approval of the Institutional Review Board.
When available: With publication

Supporting Documents
Document types: None

Additional Information
Who can access the data: Prof Woon Puay Koh (kohwp@nus.edu.sg) and Prof An Pan (panan@hust.edu.cn).
Types of analyses: Data analysis proposal for meta-analysis.
Mechanisms of data availability: The proposal will be evaluated by the principal investigators and the Institutional Review Board.
Any additional restrictions: No commercial use.
